# Purification and structure of luminal domain C of human Niemann–Pick C1 protein

**DOI:** 10.1107/S2053230X23000705

**Published:** 2023-02-02

**Authors:** Laura Odongo, Kaneil K. Zadrozny, William E. Diehl, Jeremy Luban, Judith M. White, Barbie K. Ganser-Pornillos, Lukas K. Tamm, Owen Pornillos

**Affiliations:** a Center for Membrane and Cell Physiology, University of Virginia, Charlottesville, VA 22908, USA; b Department of Molecular Physiology and Biological Physics, University of Virginia, Charlottesville, VA 22908, USA; c Program in Molecular Medicine, University of Massachusetts Medical School, Worcester, MA 01605, USA; d Department of Cell Biology, University of Virginia, Charlottesville, VA 22908, USA; University of Toronto, Canada

**Keywords:** Niemann–Pick C1 protein, Ebola virus, cholesterol, membrane proteins

## Abstract

Niemann–Pick C1 protein (NPC1) is a membrane protein that primarily resides in late endosomes and lysosomes, and plays an important role in cholesterol homeostasis in the cell. The second luminal domain of NPC1 (NPC1-C) serves as the intracellular receptor for Ebola and Marburg viruses. Here, the recombinant production of nonglycosylated and glycosylated NPC1-C and a new crystal form of the nonglycosylated protein are reported. The crystals belonged to space group *P*2_1_ and diffracted to 2.3 Å resolution. The structure is similar to other reported structures of NPC1-C, with differences observed in the protruding loops when compared with NPC1-C in complex with Ebola virus glycoprotein or NPC2.

## Abstract 2

New purification protocols for the second luminal domain of Nieman–Pick C1 protein, which serves as the intracellular receptor for Ebola and Marburg viruses, are reported together with a crystal structure, offering a structural view of the Ebola virus binding site.

## 1. Introduction

Cholesterol is delivered to cells by low-density lipoprotein (LDL) particles, which contain the esterified form of cholesterol. LDL is transported to lysosomes, where it encounters acid hydrolases that convert cholesteryl ester to cholesterol (Goldstein *et al.*, 1975 ▸). The cholesterol is then trafficked to the plasma membrane and endoplasmic reticulum (Storch & Xu, 2009 ▸). Niemann–Pick C1 (NPC1) and C2 (NPC2) are two endosomal proteins that are involved in cholesterol transport (Storch & Xu, 2009 ▸). NPC1 is a 13-pass transmembrane glycoprotein that resides primarily in late endosomes and lysosomes. NPC1, along with the other membrane proteins in lysosomes, is highly glycosylated, forming the glycocalyx, which is thought to protect the limiting membrane from hydrolytic degradation (Rudnik & Damme, 2021 ▸). NPC1 is proposed to regulate cholesterol egress by facilitating transit of cholesterol through the glycocalyx (Kwon *et al.*, 2009 ▸; Li *et al.*, 2015 ▸) as well as regulating endoplasmic reticulum contact sites with late endosomes (Höglinger *et al.*, 2019 ▸). Loss-of-function mutations in NPC1 or NPC2 result in Niemann–Pick type C disease (Evans & Hendriksz, 2017 ▸). This disease is characterized by the accumulation of sphingosine, which causes dysregulation of calcium homeostasis, which in turn results in the secondary storage of cholesterol and sphingo­lipids (Lloyd-Evans *et al.*, 2008 ▸).

NPC1 is also a critical host factor for filovirus entry and infection, serving as an obligate endosomal receptor (Côté *et al.*, 2011 ▸; Carette *et al.*, 2011 ▸). Filoviruses, including Ebola and Marburg viruses, cause hemorrhagic fever associated with fatality rates of up to 90%. The viral glycoprotein (GP) interacts with domain C of NPC1 (termed NPC1-C here), which is one of three large luminal loops of the protein. NPC1-C is minimally sufficient for interaction with GP, but all three luminal domains of NPC1 are required for efficient viral entry (Miller *et al.*, 2012 ▸). Viral entry is not dependent on the cholesterol transport activity (Côté *et al.*, 2011 ▸; Carette *et al.*, 2011 ▸).

In this study, we report detailed methods for the purification of NPC1-C and report its X-ray crystal structure at 2.3 Å resolution.

## 2. Methods

### 2.1. Protein expression and purification

#### 2.1.1. Production of nonglycosylated NPC1-C

Human NPC1-C (residues 371–621) was expressed using pET-41a vector in *Escherichia coli* BL21(DE3) cells with a C-terminal His_8_ tag. The cells were grown to an OD_600_ of 0.6–0.8 and protein production was induced with 0.5 m*M* isopropyl β-d-thiogalactopyranoside for 4 h at 30°C. The cells were harvested by centrifugation (3700*g* at 4°C for 15 min) and were stored at −80°C. To purify the protein, the cell pellet from 0.5 l of bacterial culture was thawed and resuspended in 50 ml lysis buffer (20 m*M* Tris pH 8.0, 300 m*M* NaCl, 5 m*M* β-mercaptoethanol) supplemented with protease-inhibitor cocktail (Thermo Scientific). The cells were lysed by sonication and the cell lysate was centrifuged at 18 000*g* at 4°C for 30 min. The pellet was resuspended in 50 ml solubilization buffer (8 *M* urea, 20 m*M* Tris pH 8.0, 300 m*M* NaCl, 5 m*M* β-mercaptoethanol) and incubated at room temperature for 30 min. The suspension was then centrifuged at 18 000*g* at 10°C for 30 min. The supernatant was mixed with 4 ml Ni–NTA beads (Thermo Scientific) pre-equilibrated with solubilization buffer and was incubated overnight on a rotator at room temperature. The beads were then washed with 100 ml solubilization buffer. The protein was eluted using elution buffer (8 *M* urea, 20 m*M* Tris pH 8.0, 300 m*M* NaCl, 200 m*M* imidazole, 5 m*M* β-mercaptoethanol). To refold the protein, sequential dialysis was performed at 4°C: 2 l buffer *A* (4 *M* urea, 50 m*M* Tris pH 8.0, 200 m*M* NaCl) for 10–12 h, 2 l buffer *B* (2 *M* urea, 50 m*M* Tris pH 8.0, 100 m*M* NaCl) overnight, 500 ml refolding buffer (100 m*M* Tris pH 8.0, 400 m*M* l-Arg, 5 m*M* reduced glutathione, 0.5 m*M* oxidized glutathione) for 2 h and finally 2 l dialysis buffer *C* (20 m*M* Tris pH 8.0) for 2 × 24 h. The sample was then concentrated using a filter cell (10 000 molecular-weight cutoff filter) and mixed with an equal volume of size-exclusion chromatography (SEC) buffer (20 m*M* MES pH 5.5, 100 m*M* NaCl). Precipitate was removed by centrifugation (4000*g* at 4°C for 20 min). The supernatant was then concentrated to 2–3 ml in a 10 000 molecular-weight cutoff Amicon filter (MilliporeSigma) and further purified by size-exclusion chromatography on a Superdex 200 16/60 column (GE Healthcare) equilibrated with SEC buffer. Fractions containing the target protein were pooled and concentrated. The concentrated protein was stored at −80°C. Our purification protocol reproducibly yielded around 2 mg of pure, folded NPC1-C per litre of *E. coli* culture.

#### 2.1.2. Production of glycosylated NPC1-C

A pDisplay plasmid encoding human NPC1-C (Val372–Val621) with an N-terminal His_6_ tag, with the NPC1 residues flanked by sequences that form a coiled coil (Deffieu & Pfeffer, 2011 ▸), was used. 293-F cells were seeded in 125 ml shaker flasks (1 × 10^6^ cells ml^−1^) and transfected with the plasmid using 293fectin reagent with or without 5 µ*M* kifunensine. 24 h post-transfection, 10 m*M* sodium butyrate was added to induce protein expression. 72 h post-transfection, the cell suspension was collected and clarified by centrifugation (2400*g*, 4°C, 5 min). The clarified medium was filtered (0.2 µm), supplemented with protease-inhibitor cocktail and concentrated using Amicon Ultra-15 (30 000 molecular-weight cutoff). The concentrated protein solution was then added to 5 ml Ni-Sepharose beads that had been pre-equilibrated with binding buffer (50 m*M* MES pH 6.5, 150 m*M* NaCl, 20 m*M* imidazole) and incubated overnight at 4°C with gentle agitation. After washing, the protein was eluted using elution buffer (50 m*M* MES pH 6.5, 150 m*M* NaCl, 300 m*M* imidazole), dialyzed three times against 1 l dialysis buffer (50 m*M* MES pH 5.5, 150 m*M* NaCl), concentrated using an Amicon filter (30 000 molecular-weight cutoff) and stored at −80°C.

#### 2.1.3. Production of glycosylated A82V Ebola virus (EBOV) glycoprotein (GP)

The A82V EBOV GP construct lacked the mucin-like and transmembrane domains (residues 312–462 and 633–676), and one N-linked glycosylation site (T42V) was mutated (Lee *et al.*, 2008 ▸). Recombinant baculoviruses expressing A82V EBOV GP with a C-terminal One-STrEP-FLAG tag were generated using the Bac-to-Bac baculovirus expression system (Thermo Scientific). Suspension Sf9 insect cells grown in ESF-921 medium (Expression Systems) were infected with recombinant baculoviruses at a multiplicity of infection of 10 (Hanson *et al.*, 2007 ▸). 72 h after infection, the cell supernatant was collected, filtered and concentrated using a Sartorius tangential flow filtration system with a 10 000 molecular-weight cutoff filter (Sartorius). The pH of the concentrate was adjusted to pH 7.5 with 1 *M* Tris pH 9 and it was clarified by centrifugation (38 400*g*, 30 min). The concentrate was further filtered using a PES 0.22 µm filtration unit. The filtered concentrate was then loaded onto StrepTactin beads (IBA Lifesciences) that had been pre-equilibrated in phosphate-buffered saline (PBS) and was incubated overnight with gentle agitation. The StrepTactin–supernatant slurry was loaded into a 1.5 × 20 cm glass chromatography column (Bio-Rad). Following column loading, the flowthrough was passed through the column a second time and subsequently washed extensively (three column volumes) with PBS; the protein was then eluted with 10 m*M* desthiobiotin in PBS. The eluate was first concentrated using an Amicon filter (50 kDa molecular-weight cutoff) and the buffer was then exchanged to HMSS (thermolysin) buffer [20 m*M* HEPES, 20 m*M* MES, 150 m*M* NaCl, 10%(*w*/*v*) sucrose pH 7.9]. The protein was cleaved with 1 mg ml^−1^ thermolysin (MilliporeSigma; 1:50 enzyme:substrate by mass) in the presence of 50 m*M* CaCl_2_ to generate primed (∼19 kDa) A82V EBOV GP. Proteolysis was allowed to proceed for 18–24 h at 4°C and the reaction was terminated by the addition of 10 m*M* phosphoramidon (MilliporeSigma). The cleaved protein was immediately applied onto a Superdex 200 column (Santa Cruz) pre-equilibrated with HMS buffer (20 m*M* HEPES, 20 m*M* MES, 130 m*M* NaCl pH 7.5). Fractions corresponding to primed A82V EBOV GP were pooled, concentrated using an Amicon filter (30 000 molecular-weight cutoff) and stored at −80°C. Following collection, all steps were performed at 4°C.

### 2.2. Crystallization and structure determination

Crystallization trials were performed by mixing 5 mg ml^−1^ NPC1-C with 5 mg ml^−1^ primed A82V EBOV GP in an attempt to co-crystallize the complex. Crystals were grown at 20°C in sitting drops consisting of equal volumes of protein solution and precipitant [0.1 *M* sodium malonate pH 6, 20%(*w*/*v*) PEG 3350] for at least three days. The crystals were cryoprotected with 20%(*v*/*v*) glycerol and flash-cooled in liquid nitrogen. Diffraction data were collected on beamline 22-ID at the Advanced Photon Source (APS). Indexing, integration and scaling were performed with *HKL*-2000 (Otwinowski & Minor, 1997 ▸). Although diffraction spots were observed beyond 2.3 Å resolution, the data were truncated at this resolution to ensure high completeness and keep CC_1/2_ in the last resolution shell above 0.2. The structure was solved by molecular replacement with *MOLREP* (Vagin & Teplyakov, 2010 ▸) using PDB entry 5f18 as the search model. Iterative refinement, model building and validation were performed using *Phenix* (Adams *et al.*, 2011 ▸) and *Coot* (Emsley *et al.*, 2010 ▸). Structure validation was performed throughout the refinement process using *MolProbity* (Chen *et al.*, 2010 ▸) as implemented in *Phenix*. Figures were prepared using *PyMOL* (Schrödinger).

## 3. Results and discussion

### 3.1. Production of nonglycosylated NPC1-C

**Figure 1:**
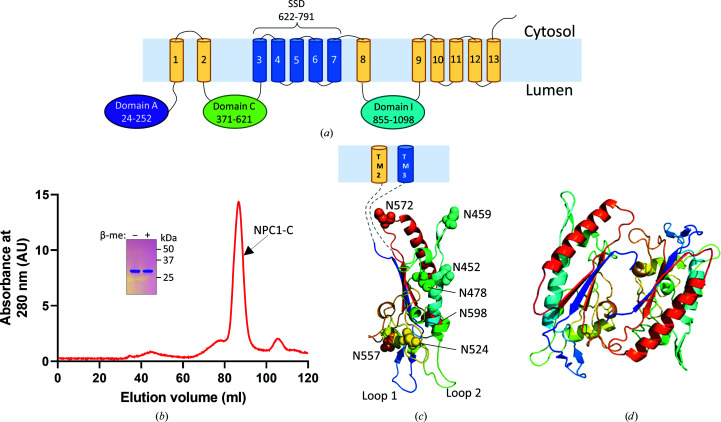
(*a*) A schematic of NPC1 showing the 13 transmembrane domains (1–13), luminal domains A, C and I and the sterol-sensing domain (SSD). (*b*) Ni–NTA affinity-purified His_8_-tagged NPC1-C was refolded and purified to homogeneity using size-exclusion chromatography. A representative Superdex 200 16/60 gel-filtration profile of the refolded protein is shown. The expected molecular weight of NPC1-C is 30 kDa. The inset shows SDS–PAGE profiles of the final purified protein taken from the labeled peak and prepared under nonreducing (β-me −) and reducing (β-me +) buffer conditions. (*c*) Cartoon representation of an NPC1-C monomer, rainbow-colored from the N-­terminus (blue) to the C-terminus (red), with the seven predicted N-linked glycosylation sites (Asn452, Asn459, Asn478, Asn524, Asn557, Asn572 and Asn598) indicated as spheres. The dashed lines indicate linker segments between NPC1-C and transmembrane domains 2 and 3 (TM2 and TM3). (*d*) Antiparallel dimer in the asymmetric unit.

The goal was to co-crystallize NPC1-C in complex with primed A82V Ebola virus glycoprotein (GP) as part of an effort to explore the basis of the enhanced infectivity of the A82V variant in the 2013–2016 Ebola outbreak in West Africa (Diehl *et al.*, 2016 ▸; Urbanowicz *et al.*, 2016 ▸; Wang *et al.*, 2017 ▸). Human NPC1-C (residues 371–621; Fig. 1 ▸ *a*) was expressed as a C-terminally polyhistidine-tagged fusion protein in *E. coli* as insoluble inclusion bodies. After denaturation with urea and a nickel-affinity chromatography step, the protein was refolded at pH 8 using the glutathione redox system to facilitate correct disulfide-bond formation (De Bernardez Clark *et al.*, 1999 ▸; Mayer & Buchner, 2004 ▸). Since NPC1 is an endolysosomal protein, where the pH range is 4.5–5.5, buffer exchange was performed to decrease the pH to 5.5. Additionally, more protein aggregation was observed at pH 8 than at pH 5.5. The precipitate that formed upon changing the pH from 8 to 5.5 was removed by centrifugation. The supernatant was then concentrated and applied onto a size-exclusion column. The protein eluted as a single major species, with SDS–PAGE revealing a pure protein with the expected apparent molecular mass of approximately 30 kDa (Fig. 1 ▸ *b*). Additional smaller peaks observed in the chromatogram are likely to be aggregates of NPC1-C or low levels of impurities.

### 3.2. Crystal structure of NPC1-C

We set up screens with nonglycosylated NPC1-C as well as with glycosylated NPC1-C (from 293F cells) with primed and glycosylated A82V EBOV GP (Chandran *et al.*, 2005 ▸; Schornberg *et al.*, 2006 ▸). To aid the crystallization efforts, we also prepared glycosylated NPC1-C from kifunensine-treated cells. Kifunensine is a mannosidase inhibitor that results in the formation of a glycoprotein with short mannose-rich sugar chains (Elbein *et al.*, 1990 ▸; Nettleship *et al.*, 2009 ▸; Chang *et al.*, 2007 ▸). We obtained crystals from initial screens for all three forms of NPC1-C, but only obtained high-resolution diffraction data from samples containing nonglycosylated NPC1-C.

Although the objective was to obtain a crystal of a protein complex of NPC1-C with the primed and glycosylated form of A82V EBOV GP, the proteins did not co-crystallize regardless of the glycosylation state of NPC1-C, with nonglycosylated NPC1-C crystallizing alone. After molecular replacement, the modeled coordinates were refined against data extending to 2.3 Å resolution (Table 1 ▸). NPC1-C crystallized in space group *P*2_1_ (Fig. 1 ▸ *c*) with two molecules in the asymmetric unit (Fig. 1 ▸ *d*). Electron densities were well defined for residues 400–606 in chain *A* and residues 401–607 in chain *C*. The two polypeptide chains packed in an antiparallel fashion in the asymmetric unit; however, the protein purified as a monomer as determined by size-exclusion chromatography (Fig. 1 ▸ *b*). Noncrystallographic dimer packing resulted in the unfolding of an N-terminal α-helix (residues 384–399) as observed in a published crystal structure of NPC1-C (PDB entry 5f18; Wang *et al.*, 2016 ▸). The two disulfide bonds that were expected in the structure, *i.e.* Cys468–Cys479 and Cys516–Cys533, are clearly resolved in the electron density, indicating that the refolding protocol was effective.

### 3.3. Comparison with other published structures of NPC1-C

**Figure 2:**
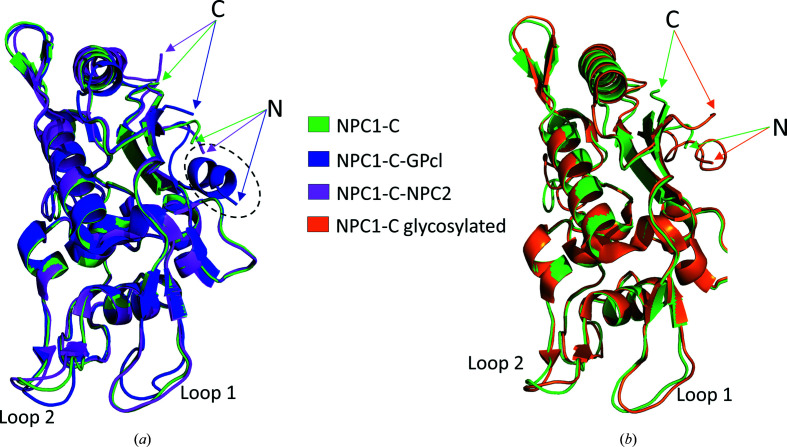
(*a*) Overlay of NPC1-C with NPC1-C–EBOV GP (PDB entry 5f1b) and NPC1-C–NPC2 (PDB entry 5kwy). The dashed oval indicates the location of the N-­terminal helix, which is unfolded in our structure. (*b*) Overlay of NPC1-C with the crystal structure of the glycosylated form of NPC1-C (PDB entry 5hns). The N- and C-termini are labeled N and C, respectively.

We compared our structure of nonglycosylated NPC1-C with published structures of apo nonglycosylated NPC1-C (PDB entry 5f18; Wang *et al.*, 2016 ▸), apo glycosylated NPC1-C (PDB entry 5hns; Zhao *et al.*, 2016 ▸), NPC1-C in complex with NPC2 (PDB entry 5kwy; Li *et al.*, 2016 ▸), NPC1-C in complex with primed EBOV GP (PDB entry 5f1b; Wang *et al.*, 2016 ▸) and the full-length structure of NPC1 (PDB entry 6w5r; Qian *et al.*, 2020 ▸). The overall structure of NPC1-C is similar to other published structures of NPC1, as expected, with root-mean-square deviations on all common C^α^ atoms for each aligned pair ranging from 0.36 to 0.78 Å. In full-length NPC1, domains C and I fold into similar structures, intertwine with each other through an extensive interface and form a tunnel that may function as a path for cholesterol transfer (Qian *et al.*, 2020 ▸). It is likely that the absence of domain I allowed NPC1-C to crystallize as dimers using the same domain I packing interface. Of note, the crystal structure of apo glycosylated NPC1-C (PDB entry 5hns) also crystallized as an apparent dimer (Zhao *et al.*, 2016 ▸). While another structure of apo non­glycosylated NPC1-C (PDB entry 5f18) did not crystallize as a dimer, some of the crystal-packing contacts also formed along the domain I-interacting interface (Wang *et al.*, 2016 ▸). The main differences between our structure and NPC1-C in complex with primed EBOV GP (Wang *et al.*, 2016 ▸) or NPC2 (Li *et al.*, 2016 ▸) is in the configurations of protruding loops (loop 1, Tyr420–Asp428; loop 2, Asp501–Tyr506; shown in Figs. 2 ▸ *a* and 2 ▸ *b*). NPC1-C uses these loops to engage with its binding partners, explaining the different configurations. In another apo NPC1-C structure (PDB entry 5f18), both loops are engaged in crystal contacts (Wang *et al.*, 2016 ▸). Our structure provides further support for the conclusion that the luminal C domain is a stably folded interaction module of NPC1.

NPC1-C has seven predicted N-glycosylation sites: Asn452, Asn459, Asn478, Asn524, Asn557, Asn572 and Asn598 (Fig. 1 ▸ *c*; Zhao *et al.*, 2016 ▸; Gong *et al.*, 2016 ▸). N-glycosylation does not affect the binding to primed EBOV GP (Ndungo *et al.*, 2016 ▸; Wang *et al.*, 2016 ▸); consistent with this, comparison of our nonglycosylated structure with that of glycosylated NPC1-C (PDB entry 5hns) does not indicate large conformational changes. Currently, the known role of glycosylation of NPC1 is to contribute to the glycocalyx, which protects the membrane proteins and lipids from hydrolytic enzymes (Rudnik & Damme, 2021 ▸).

Interestingly, noncrystallographic packing of the two copies of NPC1-C in our crystal resulted in the unfolding of an N-terminal α-helix (residues 384–399) as also observed in the apo nonglycosylated form of NPC1-C (PDB entry 5f18); this α-helix is partially folded in the structure of the NPC1-C–EBOV GP complex (PDB entry 5f1b; Wang *et al.*, 2016 ▸; dashed oval in Fig. 1 ▸ *c*). This helix is also not resolved in other published structures of NPC1-C (Li *et al.*, 2016 ▸). This result may indicate the dynamic nature of this portion of NPC1-C, which is connected to one of the transmembrane segments that undergo conformational rearrangements during cholesterol transport (Qian *et al.*, 2020 ▸).

## 4. Conclusion

The results of our structural study add to the existing body of work on NPC1 as well as providing detailed protocols for the efficient production of recombinant NPC1-C expressed in *E. coli* and mammalian cells. Domain C of NPC1 is an independently folding module with flexible protruding loops that interact with different binding partners, including EBOV GP and NPC2 (Wang *et al.*, 2016 ▸; Li *et al.*, 2016 ▸).

## Supplementary Material

PDB reference: NPC1 luminal domain C, 8eus

## Figures and Tables

**Table 1:** Structure-determination statistics Values in parentheses are for the highest resolution shell.

Data collection	
Beamline	22-ID, APS
Wavelength (Å)	1.000
Data-processing program	*HKL*-2000
Space group	*P*2_1_
*a*, *b*, *c* (Å)	53.145, 65.207, 68.534
α, β, γ (°)	90, 91.686, 90
Resolution range (Å)	50–2.30 (2.37–2.30)
*R*_meas_	0.133 (0.677)
*R*_p.i.m._	0.053 (0.302)
CC_1/2_	0.998 (0.772)
Mean *I*/σ(*I*)	13.1 (3.1)
Completeness (%)	98.2 (97.4)
Average multiplicity	5.5 (4.8)
Wilson *B* factor (Å^2^)	26.7
Phasing	
Phasing program	*MOLREP*
Method	Molecular replacement
Search model	PDB entry 5f18
Refinement	
Refinement program	*Phenix* v.1.20.1-4487 (*phenix.refine*)
Resolution range	41.2–2.30 (2.40–2.30)
No. of unique reflections	20421 (2292)
No. of reflections for *R*_free_	1088 (136)
*R*_work_	0.19 (0.25)
*R*_free_	0.23 (0.31)
No. of non-H atoms	
Protein	3343
Water	160
Average *B* factor (Å^2^)	
Protein	32.8
Water	33.8
R.m.s.d., bond lengths (Å)	0.003
R.m.s.d., bond angles (°)	0.646
Validation and deposition	
Ramachandran favored (%)	96.82
Ramachandran outliers (%)	0
Ramachandran *Z*-score	−0.09
Rotamer outliers (%)	1.08
*MolProbity* clashscore	1.38
PDB code	8eus
